# Polarization‐Selective Metamaterial Absorber for Long‐ to Very Long‐Wavelength Infrared Broadband

**DOI:** 10.1002/nap2.70130

**Published:** 2026-05-11

**Authors:** Zhe Wu, Zhongzhu Liang, Xiaoyan Shi, Fuming Yang, Siyu Guo, Jihui Jiang, Wenwen Sun, Xintong Wei, Bing Sun, David R. Smith, Haiyang Xu

**Affiliations:** ^1^ State Key Laboratory of Integrated Optoelectronics and Key Laboratory of Ultraviolet Light‐Emitting Materials and Technology of Ministry of Education, College of Physics Northeast Normal University Changchun China; ^2^ Center for Metamaterials and Integrated Plasmonics, Department of Electrical and Computer Engineering Duke University Durham North Carolina USA

## Abstract

Metamaterial absorbers have received extensive research interest and sustained attention due to their excellent resonance characteristics, including ultrathinness, lightweight structure, and high light absorption. The broadband polarization‐sensitive metamaterial absorber based on surface plasmon polaritons has the advantages of high energy utilization efficiency and good stability, making it a potential substitute for traditional polarization devices. This study demonstrates the polarization‐selective absorption mechanism of Cu/Si/Ti three‐layer absorbers for long‐wavelength infrared light, achieving an extinction ratio of 14. Subsequently, a four‐structure composite‐material surface pattern design was proposed, which achieved an average extinction ratio of 9 in the range of 8–14 μm through various localized surface plasmon resonance coupling mechanisms. By further utilizing nested composite structures to extend the operating range to the very long‐wavelength range, an average extinction ratio of 17 was achieved in the range of 16–30 μm. This series of designs provides a new method for broadband polarization‐selective absorbers by adjusting the excitation conditions of localized surface plasmon resonance. The proposed absorber can be integrated into the focal plane of infrared detectors to achieve polarization‐sensitive infrared imaging, with enormous potential applications in military reconnaissance, environmental monitoring, biosensing, and other fields.

## Graphical abstract

Broadband and polarization‐selective metamaterial absorbers for long‐ to very long‐wavelength infrared light with high extinction ratios are designed and demonstrated, which provide a new method for broadband polarization‐selective absorbers.

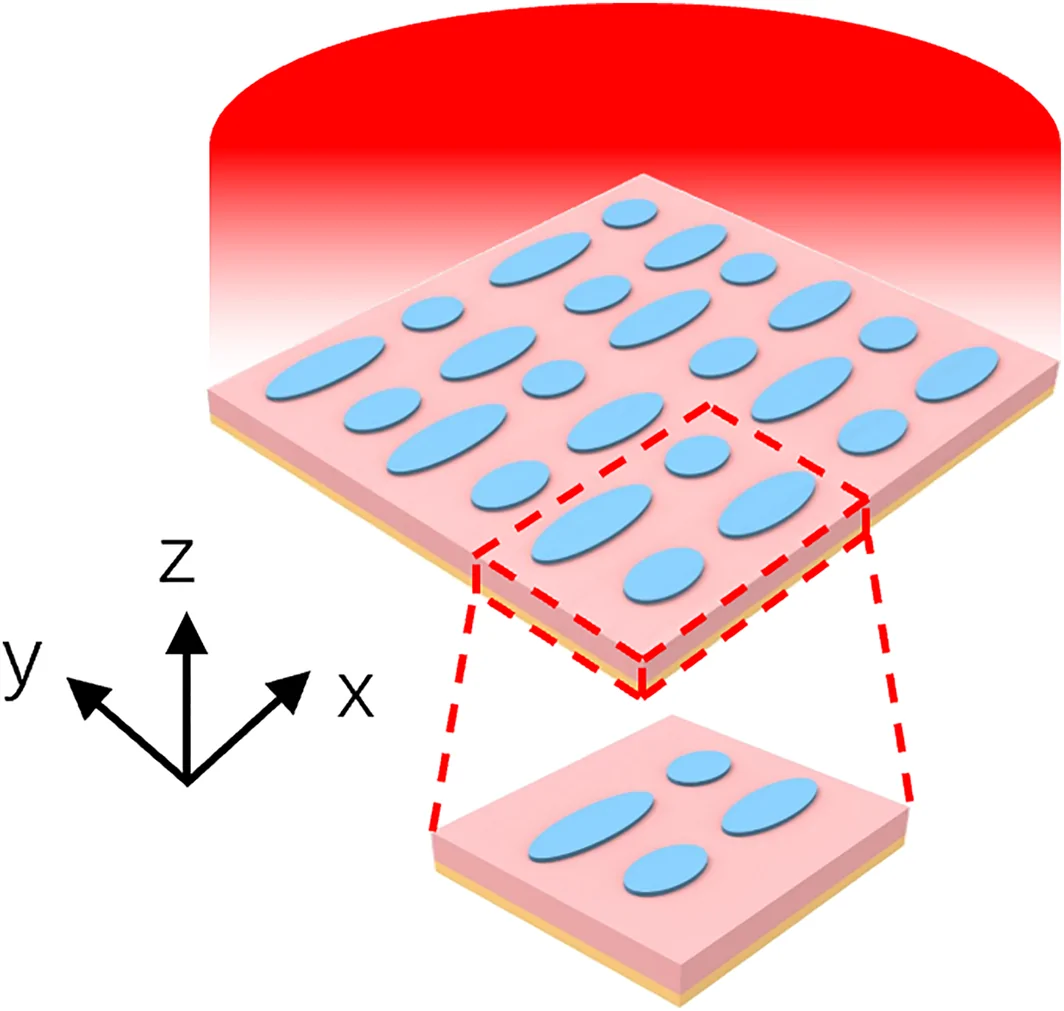

## 1 Introduction

Broadband polarization‐sensitive metamaterial absorbers, as potential alternatives to traditional polarization devices, can be integrated into the focal plane of infrared detectors to achieve polarization‐sensitive infrared imaging. They have enormous potential applications in military reconnaissance, environmental monitoring, biosensing, and other fields [1, 2, 3, 4, 5, 6, 7, 8, 9, 10]. In the infrared region, especially in the long‐wave infrared (LWIR) and very long‐wave infrared (VLWIR) atmospheric windows, polarization analysis is essential for enhancing contrast under low‐visibility conditions and identifying artificial objects against natural backgrounds [11, 12, 13]. This capability is crucial for applications such as missile warning systems, satellite tracking, and geological surveys [14, 15, 16]. Traditional infrared polarization detection systems typically rely on bulky optical components such as wire‐grid polarizers or birefringent crystals, which are physically separated from detector pixels and inevitably introduce alignment errors, optical cross talk, and reduced system stability. The polarizer itself generates significant energy loss, resulting in low photon utilization efficiency [17, 18, 19, 20].

Metamaterials, with their unprecedented ability to customize light–matter interactions at subwavelength scales, have become a disruptive platform for developing ultrathin optical devices [21, 22, 23]. Polarization‐sensitive metamaterial absorbers have shown significant potential in replacing traditional polarizers by directly integrating polarization selection functions at the pixel level [24, 25, 26, 27]. This integration can significantly miniaturize optical systems and improve detection performance. However, a major challenge still exists; most reported metamaterial absorbers achieve polarization selectivity at the expense of operating bandwidth, and their resonance characteristics often lead to narrowband absorption, which severely limits energy efficiency and signal‐to‐noise ratio in practical applications that require broad spectral coverage [28, 29]. Although strategies such as multi‐resonance superposition have been explored to expand the bandwidth, these methods often lead to increased structural complexity and manufacturing challenges, especially when pushing the operating frequency band toward the technically important but challenging VLWIR region [30, 31, 32].

This paper addresses this challenge by introducing a design strategy based on the synergistic coupling of multiple localized surface plasmon resonances. We first reveal the polarization selectivity mechanism in the Cu/Si/Ti three‐layer absorber, achieving the basic performance of polarization‐selective absorption with an extinction ratio of 14. Subsequently, by strategically combining structures of different geometric shapes, discrete localized surface plasmon resonance peaks were coupled into a wide absorption band in the long‐wave infrared region, achieving an average extinction ratio of 9 in the 8–14‐μm wavelength range. Furthermore, we successfully extended broadband polarization‐selective absorption to very long‐wavelengths through the design of nested structures, achieving an average extinction ratio of 17 in the range of 16–30 μm. This work provides a new approach for the design of broadband polarization‐selective absorbers, which has potential applications in military reconnaissance, environmental monitoring, biosensing, and other fields.

## 2 Results and Discussion

To reveal the basic principles and design strategies of polarization‐selective broadband absorption, we have designed a polarization‐selective broadband absorber with long‐ to very long‐wavelengths. The schematic diagram of the polarization‐selective metamaterial absorber is shown in Figure 1a. The absorber is a three‐layer metamaterial structure consisting of an elliptical structure array, a Si dielectric layer, and a Cu underlying metal plate. The primitive cell of the elliptical structure of the metasurface is centrally asymmetric, which results in polarization‐selective absorption characteristics. Meanwhile, the structural parameters of the original cell are *a* = 1.6 μm, *b* = 1 μm, *t*_1_ = 100 nm, *t*_2_ = 400 nm, *t*_3_ = 300 nm, and *P* = 2 μm. The design of the surface pattern structure is manufactured by maskless laser direct‐writing lithography technology, and the scanning electron microscope (SEM) image of the prepared structure is shown in Figure 1b. The thickness of the Cu substrate is greater than its skin depth in the long‐wave to very long‐wave infrared region, acting as a reflective mirror. Therefore, the transmittance of the overall device is 0, which can be ignored, and the absorption rate can be calculated as *A* = 1−*R*, where *R* is the reflectance.

**FIGURE 1:**
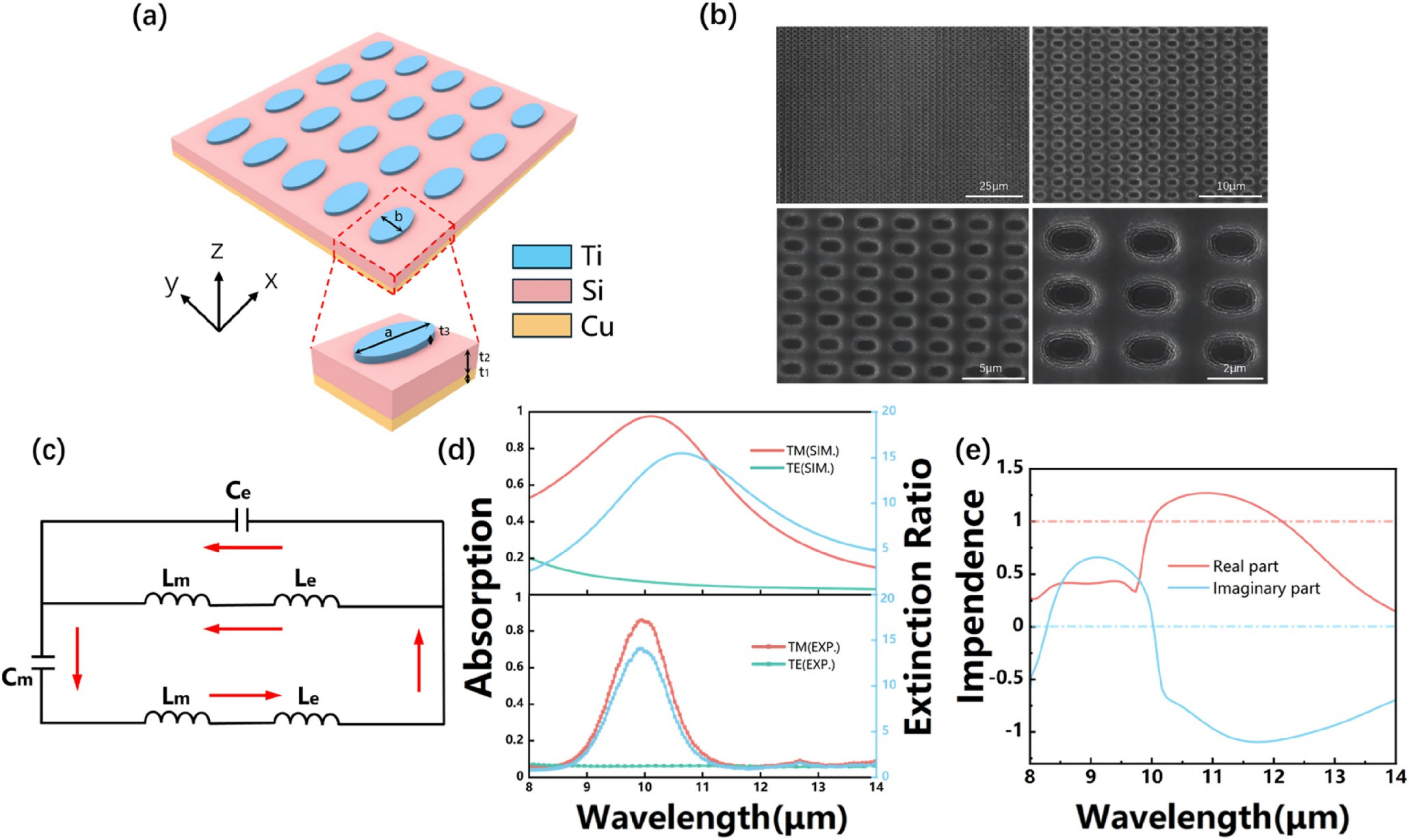
(a) Schematic diagram of the polarization‐selective metamaterial absorber. (b) SEM image of the prepared structure. (c) Schematic of the equivalent LC circuit for the LSPR mode. (d) Simulated and experimental absorption and extinction spectra for polarization‐selective absorbers. (e) Impedance of the absorber.

The existing broadband absorption strategy for metamaterials mainly combines propagating surface plasmon resonance (PSPR) and localized surface plasmon resonance (LSPR) for broadband absorption. This coupling method enables metamaterial devices to expand their absorption bandwidth and achieve efficient absorption in the broadband ranges of visible light, near‐infrared, mid‐wave infrared [33], LWIR, and VLWIR [34]. The influence of the geometric parameters of the absorber on its PSPR can be expressed as follows [35]:

(1) $$\lambda_{\text{PSP}}=P\sqrt{\frac{\varepsilon_{m}\varepsilon_{d}}{\varepsilon_{m}+\varepsilon_{d}}},$$

where *p* is the period of the absorber, and *ε*_*m*_ and *ε*_*d*_ represent the permittivities of the metal and dielectric, respectively.

As shown in Figure 1c, the LSPR mode can be explained by the equivalent circuit theory [36]:

(2) $$\lambda_{\text{LSP}}=2\pi c\sqrt{LC/2},$$

where *L* is the equivalent inductance of the absorber, which comprises two parts:

(3) $$L=L_{m}+L_{e},$$

(4) $$L_{m}=\frac{\beta_{1}\mu_{0}t_{2}}{2},$$

(5) $$L_{e}=\frac{1}{\delta \omega^{2}\varepsilon_{0}}\left(\frac{{\varepsilon_{m}^{\prime }}^{2}}{\varepsilon_{m}^{\prime }}+{\varepsilon_{m}^{\prime \prime }}^{2}\right),$$

where *L*_*m*_ represents the inductance between the surface pattern and the metal bottom, *β*_*1*_ is the effective coefficient, and *t*_*2*_ is the thickness of the dielectric layer. *L*_*e*_ represents the contribution of drift electrons to inductance, *μ*_0_ is the vacuum permeability, *ε*_0_ is the vacuum permittivity, and *δ* represents the penetration depth of the metal. *C* is the equivalent capacitance of the absorber, consisting of two parts:

(6) $$C=C_{e}+C_{m},$$

(7) $$Cm=\frac{c_{1}\varepsilon_{0}\varepsilon_{d}A}{t_{2}n},$$

(8) $$C_{e}=\frac{c_{2}\varepsilon_{0}t_{3}a}{P-a},$$

where *C*_*m*_ represents the capacitance between the surface pattern and the bottom layer, *c*_1_ is an effective coefficient, and *A* is the effective area of the surface pattern corresponding to the metal bottom. *C*_*e*_ represents the capacitance between adjacent surface patterns, and *c*_2_ is an effective coefficient representing the effective area between adjacent surface patterns.

In order to achieve polarization‐selective absorption, we adopted the method of separating the conventional broadband metamaterial absorber's LSPR from PSPR, avoiding polarization‐independent PSPR and using LSPR with strong polarization selectivity for polarization‐selective absorption. To achieve this, we needed to minimize the excitation of PSPR that is independent of polarization response or separate the response wavelengths of PSPR and LSPR as much as possible. To achieve this goal, we designed the following structure, and the absorption and extinction spectra of the metamaterial structure are shown in Figure 1d. The simulated absorption spectrum shows an absorption rate of 97.6% at 10 μm under TM polarization. In sharp contrast, the absorption rate under TE polarization is only 7.3%, and the extinction ratio is 15. The experimental results show that under TM polarization, the absorption rate at this location is 86.0%, whereas under TE polarization, the average absorption rate is only 6.1%, and the extinction ratio is 14. The absorption bandwidth of the experimental results is smaller than that of the simulation results. Because of the differences between the simulation and experimental results, the actual refractive index deviation of the prepared Cu, Si, and Ti may lead to partial deviations in the resonance effect, and there may also be some errors in the actual preparation size, such as inaccurate film thickness control.

Based on the equivalent circuit theory mentioned above, the scattering parameters and equivalent impedance of the proposed absorber can be calculated to demonstrate the absorption response. We calculated the equivalent impedance of the absorber under the TM mode through the following equations [37]:

(9) $$Z=\sqrt{\frac{{\left(1+S_{11}\right)}^{2}-S_{21}^{2}}{{\left(1-S_{11}\right)}^{2}-S_{21}^{2}}},$$

where *Z* = *Zʹ* + *iZ*ʺ. The equivalent impedance is calculated from scattering parameters, and the impedance of air is considered to be *Z*_air_ = 1. Both the real and imaginary parts of the equivalent impedance contribute to the reflectivity of the absorber. The absorption peak appears at the wavelength where the equivalent impedance matches the air impedance. As shown in Figure 1e, the impedance at the absorption peak with a wavelength of 10 μm is *Z* = 1.06−i0.07, which is very close to the impedance of air.

To better reveal the polarization‐selective absorption mechanism of the absorber, we demonstrate its electromagnetic field distribution at the absorption peak with a wavelength of 10 μm, as shown in Figure 2a. The electric field distribution is at the interface between the surface pattern and the dielectric. In the TM mode, the electric field is strongly localized at the edge of the long‐axis direction of the surface elliptical structure, which means that surface plasmon polaritons are excited in the metamaterial absorber. The magnetic field is mainly concentrated at the boundary between the elliptical surface structure and the dielectric layer and propagates into the interior of the dielectric layer, indicating that LSPR dominates the high absorption phenomenon of the TM mode in this band. In the TE mode within the same wavelength range, the electromagnetic field is relatively evenly distributed throughout the device without any local concentration, resulting in almost no absorption and complete reflection of the device under TE mode incidence. The stability of the incident angle of the absorber is a crucial parameter in practical applications, and the oblique incidence absorption spectra of the TM mode and TE mode are shown in Figure 2b. As the incident angle increases, the absorption peak in the TM mode slightly decreases but overall remains stable, whereas the absorption in the TE mode remains almost unchanged. Overall, the polarization absorption performance of the device has good incident‐angle stability in the long‐ to very long‐wavelength range.

**FIGURE 2:**
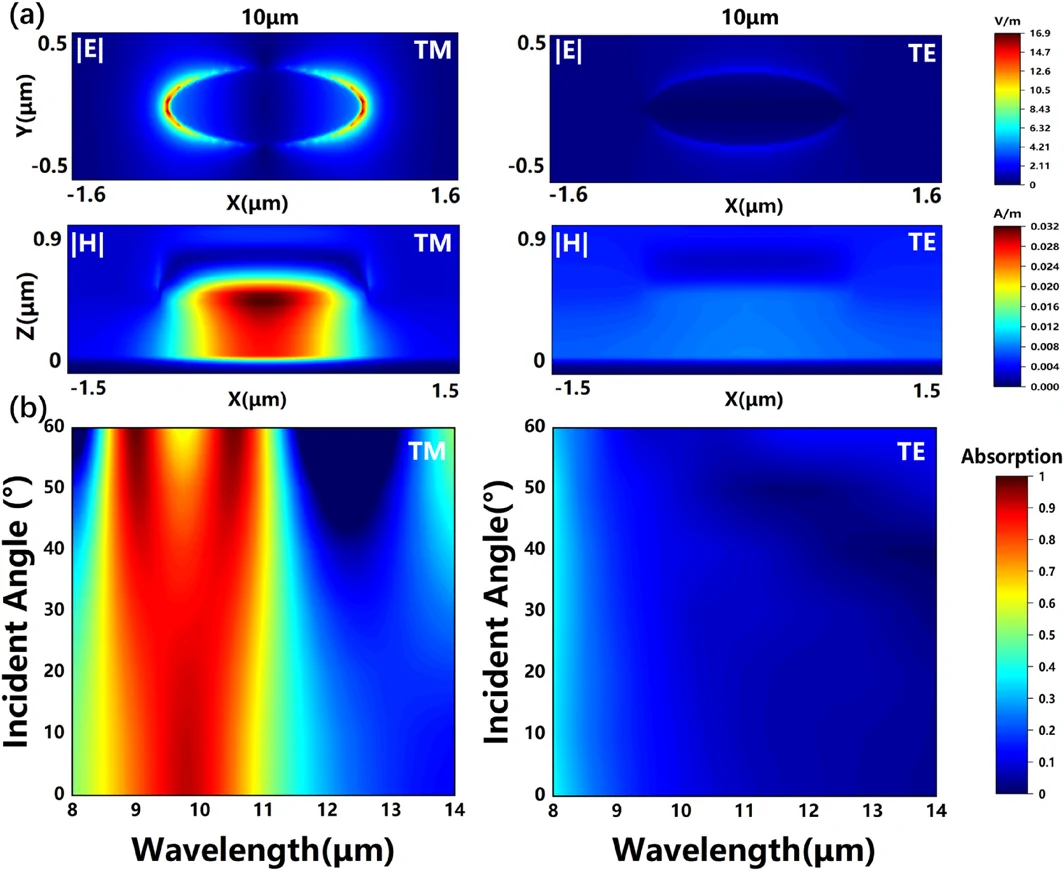
(a) Electromagnetic field distribution of the absorption peak at a wavelength of 10 μm. (b) Oblique incidence absorption spectra in TM mode and TE mode.

In order to further achieve polarization‐selective absorption of broadband, we designed a composite structure array, as shown in Figure 3a. Each cell contains four ellipses with different major‐axis sizes, arranged in a staggered manner with lengths of 1.5, 1.7, 1.9, and 2.2 μm, respectively. The short side *b* is 1 μm, *t*_1_ = 100 nm, *t*_2_ = 400 nm, *t*_3_ = 500 nm, and *P* = 5 μm. The SEM image of the prepared structure is shown in Figure 3b. The simulated and tested spectra of the designed polarization‐selective broadband absorber are shown in Figure 3c. The simulated spectrum has four absorption peaks at 9.12, 10.77, 12.29, and 14.47 μm, with absorption rates of 79.7%, 83.2%, 88.5%, and 92.4%, respectively. The average absorption rate in the TE mode is 8.52%, and the average extinction ratio is 12. Compared with the simulation results, the absorption peaks of the test results are coupled into a broad peak, with an average extinction ratio of 9. These deviations may be caused by oxidation of Ti materials, errors in film thickness, and errors in film density. The relationship between the absorption rate of the absorber and the incident polarization angle is shown in Figure 3d. As the polarization angle gradually increases from 0° (TM mode), the absorption rate of the absorber gradually decreases until the polarization angle reaches 90° (TE mode), at which point it is less than 10%, demonstrating good polarization‐selective absorption performance. Overall, the designed polarization‐selective broadband absorber exhibits excellent broadband polarization‐selective absorption performance and has potential applications in military reconnaissance, environmental monitoring, biosensing, and other fields.

**FIGURE 3:**
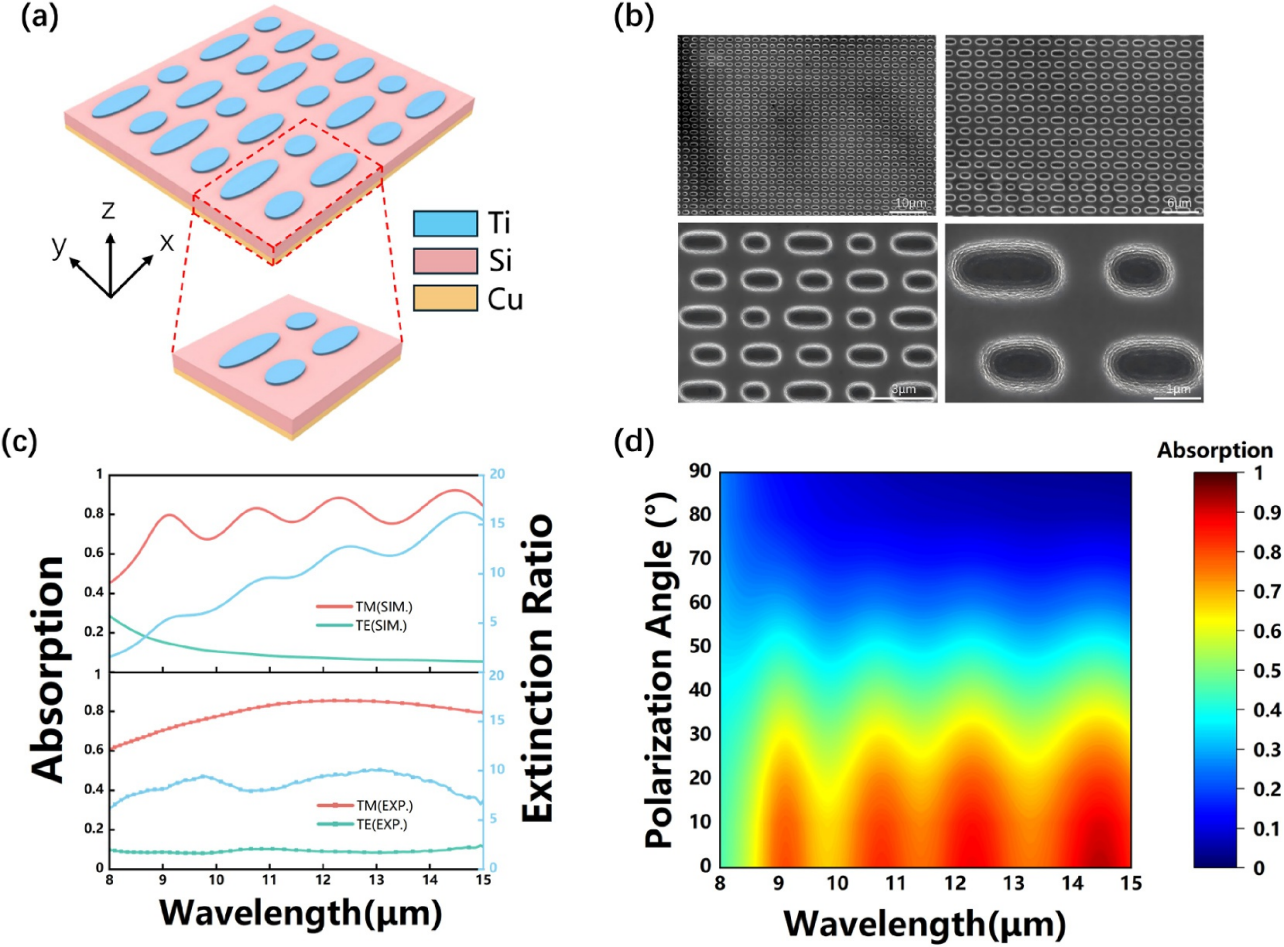
(a) Schematic diagram of the broadband polarization‐selective metamaterial absorber. (b) SEM image of the prepared structure. (c) Simulated and experimental absorption and extinction spectra for broadband polarization‐selective absorbers. (d) Absorption rate of the absorber as a function of the incident polarization angle.

To further illustrate the basic principle of polarization‐selective broadband absorption, we present the electric and magnetic field distribution diagrams at four absorption peaks with wavelengths of 9.12, 10.77, 12.29, and 14.47 μm under TM mode incidence, as shown in Figure 4a,b, respectively. The electric field distribution is located at the interface between the surface pattern and the dielectric. It is not difficult to observe that the electric fields in all four bands are strongly localized at the edges of the elliptical structures along the long‐axis direction of the corresponding sizes. From the corresponding magnetic field distributions, the magnetic fields are localized below the corresponding elliptical structures at the junction with the dielectric and propagate into the interior of the dielectric, indicating the excitation of four LSPR‐dominated absorption peaks coupled together to form broadband absorption, which also reflects the tunability of the absorber design.

**FIGURE 4:**
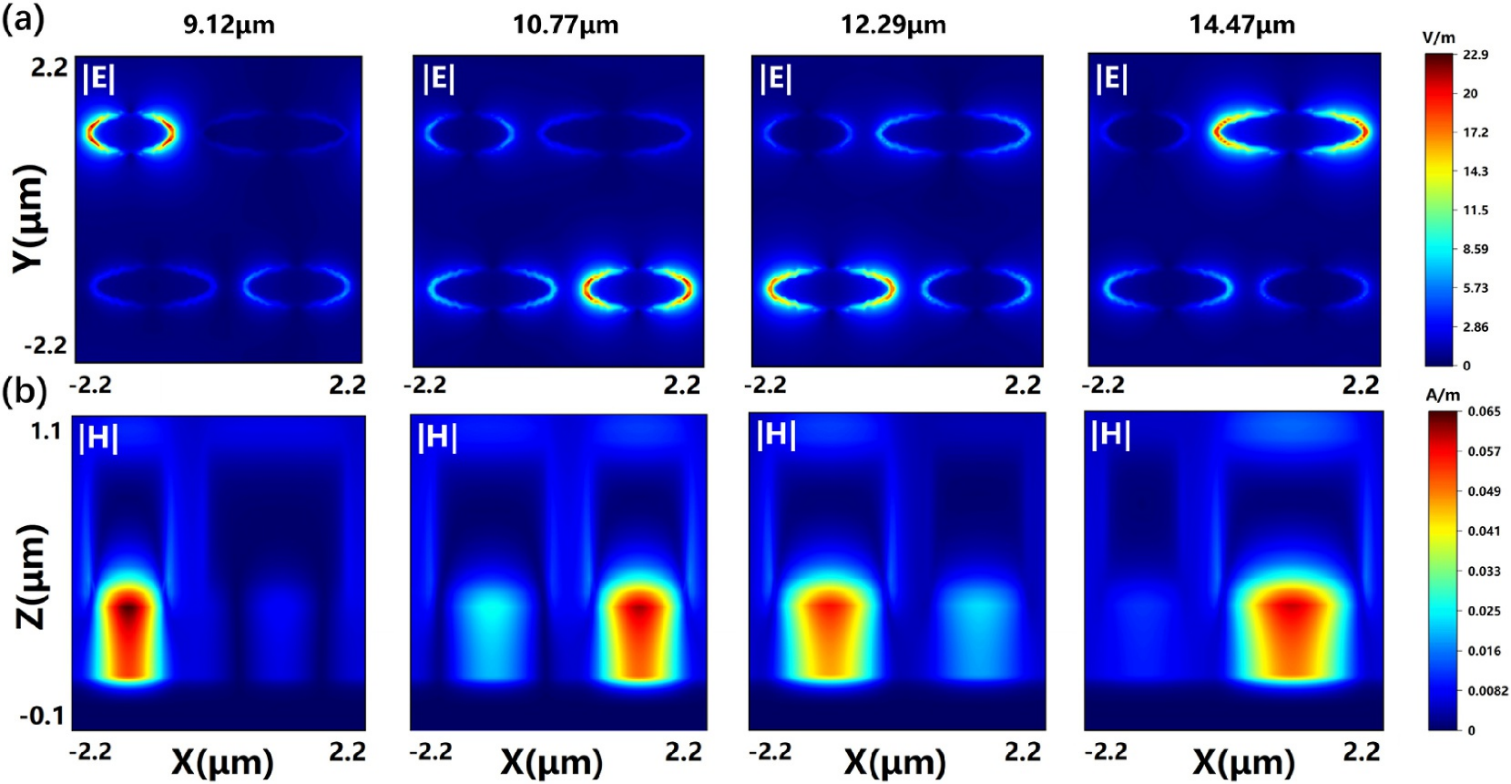
(a) Electric field distribution and (b) magnetic field distribution of four absorption peaks at wavelengths of 9.12, 10.77, 12.29, and 14.47 μm in TM mode for broadband polarization‐selective absorbers.

For imaging and detection in the very long‐wavelength infrared band, this technology plays a crucial role in interstellar target detection in space, as well as in the tracking and detection of missiles, satellites, and other space shuttles. To achieve polarization‐selective broadband absorption in the very long‐wave infrared band, we designed a new structure, and the schematic diagrams of its array and original cell are shown in Figure 5a. The surface pattern of this structure consists of an outer hollow elliptical ring nested with an inner solid ellipse, with dimensions of *a*_1_ = 1.3 μm, *a*_2_ = 1.5 μm, *a*_3_ = 1.7 μm, *a*_4_ = 1.8 μm, *a*_5_ = 2.1 μm, *a*_6_ = 2.3 μm, *b*_1_ = 0.3 μm, *b*_2_ = 0.4 μm, *b*_3_ = 0.5 μm, *t*_1_ = 100 nm, *t*_2_ = 500 nm, *t*_3_ = 400 nm, and *P* = 5 μm. The absorption and extinction spectra of the absorber are shown in Figure 5b. In the TM mode, there are two absorption peaks at wavelengths of 18.46 and 24.55 μm, with absorption rates of 52.1% and 72.5%, respectively. The average absorption rate between 16 and 30 μm is 53.1%, and the maximum extinction ratio in this band is 27, with an average value of 17. The main mechanism is to retain the effective components of the elliptical structure and couple them into broadband absorption. Table 1 shows a comparison between our designed absorber and representative studies on polarization‐selective metamaterials. It can be seen from the table that the absorber we designed has a wide operating bandwidth and a high average extinction ratio; therefore, it has high research value and a wide range of applications. Overall, the polarization‐sensitive broadband absorbers involved have excellent polarization‐selective broadband absorption performance in the long‐ to very long‐wavelength range, and have potential application prospects in the tracking and detection of missiles, satellites, and other space shuttles in military reconnaissance fields.

**FIGURE 5:**
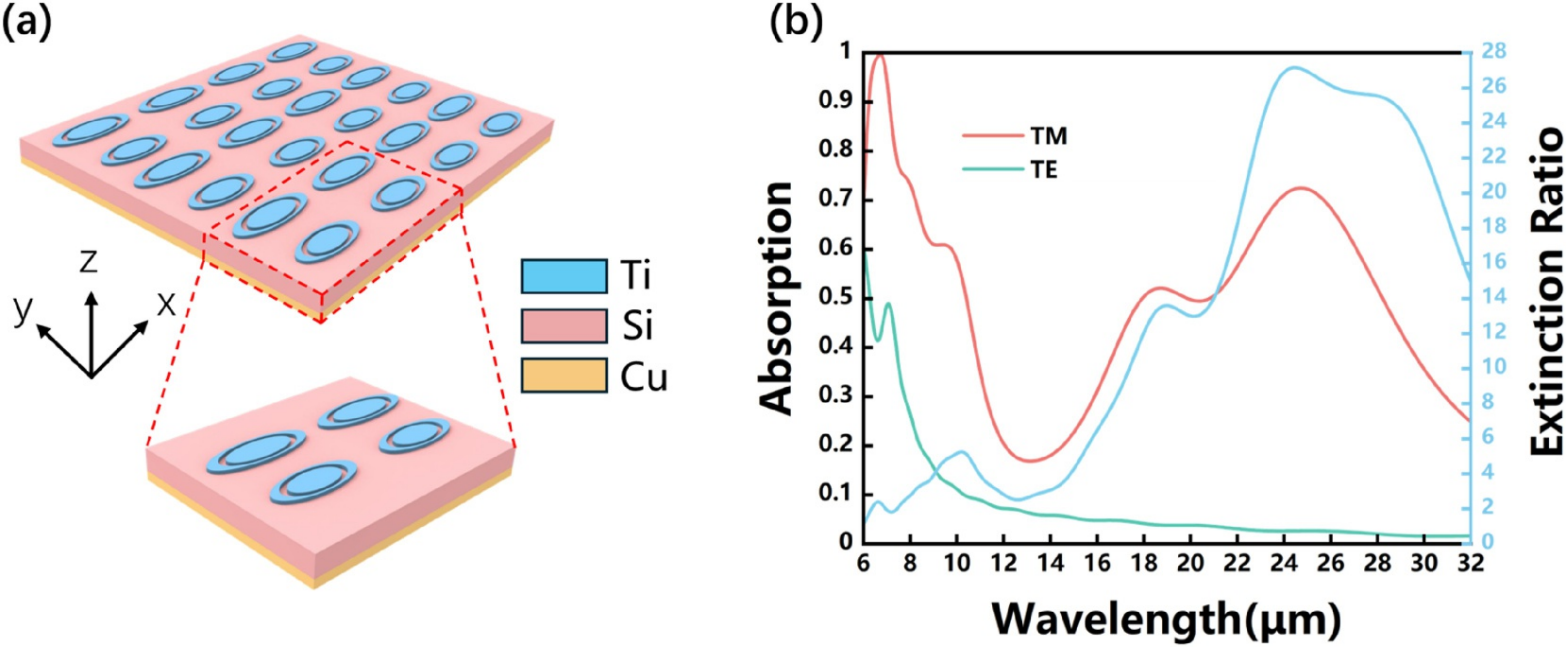
(a) Schematic diagram of the VLWIR broadband polarization‐selective metamaterial absorber. (b) Simulated absorption and extinction spectra of VLWIR broadband polarization‐selective absorbers.

**TABLE 1:** Comparison of representative studies on polarization‐selective metamaterials.

Refs.	Operation bandwidth	Extinction ratio
Hou et al. [25]	5 μm (10–15 μm)	3
Sun et al. [38]	0.2 μm (0.6–0.8 μm)	3.6
Yu et al. [24]	4 μm (8–12 μm)	4
Nguyen et al. [39]	1 μm (0.6–1.6 μm)	6.5
Shi et al. [40]	10 μm (6–16 μm)	7.9
Our work	6 μm (8–14 μm)	9
	14 μm (16–30 μm)	17

## 3 Simulations Section

Theoretical simulations were conducted on the numerical absorption spectra and electromagnetic field distribution of absorption peaks of absorbers using the finite‐difference time‐domain (FDTD) method. The unit cell of the studied structure was simulated using periodic boundary conditions along the *x*‐ and *y*‐axes and a perfectly matched layer propagating along the electromagnetic‐wave propagation direction (*z*‐axis). A grid precision of 8 was selected, the computation time was set to 5000 fs, and the minimum value for automatic shutoff was set to 1 × 10^−5^. The refractive index of Ti was obtained from the manual of the Rakic Lorentz–Drude model [41], whereas the refractive indices of Si and Cu were obtained from Shkondin [42] and Querry [43], respectively. Plane waves were emitted from the top of the element surface, and in the TM mode, the electric field propagated along the *x*‐axis direction. For oblique incidence, the incident‐angle range was 0°–60°, with a step size of 10°.

## 4 Experimental Section

This study prepared a Cu/Si/Ti structure. The substrate was silicon with a crystal orientation of 100 (TIR < 3 μm, BOW < 10 μm, and Ra < 5 nm). For substrate cleaning, the substrate was sonicated with NMP solution for 5 min, then sonicated with water for 5 min, and finally blow‐dried. Evaporative coating equipment was used to coat a 100‐nm Cu layer onto the Si substrate. For the dielectric layer of the proposed absorber, a 400‐nm Si layer was coated on top of the Cu layer. The vacuum degree during coating was less than 5 × 10^−5 ^Pa, and the coating rate was 0.5 A/s. Then, AZ5214 UV‐positive photoresist was coated onto the substrate. MLA maskless laser direct‐writing lithography was used to define the pattern of the top Ti layer. To clean the strip, the structure was completely immersed in an 80°C NMP solution. After 2 min, the Ti that needed to be peeled off visibly detached into the solution. Then, using a new solution, the structure was soaked for another 20 min and rinsed with clean water. In the end, we obtained the proposed structure.

The Fourier transform infrared spectrometer measurement system was used to demonstrate the reflection spectrum of the absorber. To determine the absolute reflectance R, the measured reflectance was completely canceled out by the opaque metal mirror in the transmission channel of the reference gold mirror, and the absorption was directly determined as *A* = 1−R based on *A* = 1‐R‐T. The measurement range was 550–2000 cm^−1^.

## 5 Conclusion

This paper reveals the polarization‐selective absorption mechanism in the long‐wave infrared region through Cu/Si/Ti three‐layer absorbers, achieving an extinction ratio of 14. A four‐structure composite‐material surface pattern design was proposed, and an average extinction ratio of 9 was obtained in the 8–14‐μm wavelength range through a multi‐LSPR coupling mechanism. By further utilizing nested composite structures to extend the operating range to the very long‐wavelength region, an average extinction ratio of 17 was achieved in the 16–30‐μm wavelength range. This series of designs reveals the mechanism of polarization selection, adjusts the excitation conditions of LSPR, preserves the effective components, and recombines them to construct a new method for designing broadband polarization‐selective absorbers. Because of its ultrathin structure and polarization‐selective absorption characteristics, the proposed absorber can be integrated into the focal plane of infrared detectors to achieve polarization‐sensitive infrared imaging, which has significant potential applications in military reconnaissance, environmental monitoring, biosensing, and other fields.

## Author Contributions

**Zhe Wu:** conceptualization, investigation, writing – original draft, writing – review and editing, visualization, validation, methodology, software, formal analysis, project administration, data curation, resources. **Zhongzhu Liang:** funding acquisition, writing – review and editing, conceptualization, methodology, visualization, formal analysis, data curation, resources, supervision, project administration. **Xiaoyan Shi:** investigation, visualization. **Fuming Yang:** investigation, visualization, formal analysis. **Siyu Guo:** investigation, visualization. **Jihui Jiang:** investigation. **Wenwen Sun:** investigation, visualization, validation, formal analysis, data curation, methodology. **Xintong Wei:** investigation, visualization. **Bing Sun:** investigation, visualization. **David R. Smith:** project administration, supervision, resources. **Haiyang Xu:** supervision, resources, project administration.

## Conflicts of Interest

The authors declare no conflicts of interest.

## Data Availability

The data that support the findings of this study are available from the corresponding author upon reasonable request.

## References

[1] H. Kim , H. Yun , S. Jeong , et al., “Optical Metasurfaces for Biomedical Imaging and Sensing,” ACS Nano (2025): 4c14751. 10.1021/acsnano.4c14751 39805079

[2] H. Lei , X. Yu , and D. Fan , “Nanocomposite Hydrogel for Real‐Time Wound Status Monitoring and Comprehensive Treatment,” Advanced Science 11, no. 42 (2024): 2405924, 10.1002/advs.202405924. PMC11558094 39269428

[3] P. Pang , W. Liu , S. Ma , et al., “Self‐Assembling Natural Flavonoid Nanomedicines for Alveolar Macrophage Reprogramming by Restoring Mitochondrial Function in Acute Lung Injury Therapy,” Chemical Engineering Journal 506 (2025): 160171, 10.1016/j.cej.2025.160171.

[4] M. Wu , H. Liu , D. Li , et al., “Smart‐Responsive Multifunctional Therapeutic System for Improved Regenerative Microenvironment and Accelerated Bone Regeneration via Mild Photothermal Therapy,” Advanced Science 11, no. 2 (2024): 2304641, 10.1002/advs.202304641. PMC10787108 37933988

[5] M. Liu , L. Qi , Y. Zou , et al., “Uncooled Near‐to Long‐Wave‐Infrared Polarization‐Sensitive Photodetectors Based on MoSe_2_/PdSe_2_ van der Waals Heterostructures,” Nature Communications 16, no. 1 (2025): 2774, 10.1038/s41467-025-58155-0. PMC11926206 40113764

[6] H. Wang , R. Lin , K. Zhao , et al., “Dual‐Way Alternation Sensing Memristors Based on (BA)_2_(MA)_3_Pb_4_Br_13_ Ferroelectric Quantum Dots With Sub‐0.16 V Switching for Broadband Image‐Recognition Systems,” Advanced Functional Materials 35, no. 46 (2025): 2510543, 10.1002/adfm.202510543.

[7] Z. Fu , J. Han , L. Wei , et al., “Polarization‐Sensitive Self‐Powered Photodetector Based on Anisotropic/Isotropic ReS_2_/SnSe_2_ van der Waals Heterojunction,” Advanced Optical Materials 12, no. 31 (2024): 2401306, 10.1002/adom.202401306.

[8] A. Hernik , J. Smets , Z. Wang , et al., “Optical Detection of Volatile Organic Compounds: A Review of Methods and Functionalized Sensing Materials,” Advanced Optical Materials 13, no. 27 (2025): e00369, 10.1002/adom.202500369.

[9] J. Rodriguez‐Alvarez , J. Vila‐Comamala , A. Garcia‐Martin , et al., “Dichroism of Coupled Multipolar Plasmonic Modes in Twisted Triskelion Stacks,” Nanophotonics 14, no. 17 (2025): 2847–2856, 10.1515/nanoph-2025-0063. PMC12397745 40896155

[10] J. Li and L. Ye , “Dielectric Dual‐Dimer Metasurface for Enhanced Mid‐Infrared Chiral Sensing Under Both Excitation Modes,” Nanophotonics 12, no. 12 (2023): 2189–2197, 10.1515/nanoph-2023-0128. PMC11501344 39634039

[11] M. Dai , C. Wang , B. Qiang , et al., “Long‐Wave Infrared Photothermoelectric Detectors With Ultrahigh Polarization Sensitivity,” Nature Communications 14, no. 1 (2023): 3421, 10.1038/s41467-023-39071-7. PMC10256712 37296149

[12] B. Qin , H. Zhu , R. Zhu , M. Zhao , M. Qiu , and Q. Li , “Space‐to‐Ground Infrared Camouflage With Radiative Heat Dissipation,” Light: Science & Applications 14, no. 1 (2025): 137, 10.1038/s41377-025-01824-y. PMC11947227 40140379

[13] Y. Xie , X. Liu , F. Li , J. Zhu , and N. Feng , “Ultra‐Wideband Enhancement on Mid‐Infrared Fingerprint Sensing for 2D Materials and Analytes of Monolayers by a Metagrating,” Nanophotonics 9, no. 9 (2020): 2927–2935, 10.1515/nanoph-2020-0180.

[14] T. Fang , K. Jiang , B. Wang , et al., “The Insertion of an Aln Spacer Between the Barrier and the Channel Layer for a Polarization‐Enhanced AlGaN‐Based Solar‐Blind Ultraviolet Detector,” ACS Applied Electronic Materials 6, no. 4 (2024): 2490–2499, 10.1021/acsaelm.4c00127.

[15] Z. Qiu , S. Wang , P. Hu , and L. Guo , “Outlier‐Robust Extended Kalman Filtering for Bioinspired Integrated Navigation System,” IEEE Transactions on Automation Science and Engineering 21, no. 4 (2024): 5881–5894, 10.1109/tase.2023.3319508.

[16] J. Meng , X. Ma , C. Chao , et al., “On the Reliability of Time Domain Induced Polarization for Contaminated Site: Partial Integral vs Full Decay,” Engineering Geology 353 (2025): 108125, 10.1016/j.enggeo.2025.108125.

[17] Z. Yang , S. Huo , Z. Zhang , et al., “High‐Precision Multibit Opto‐Electronic Synapses Based on ReS_2_/h‐BN/Graphene Heterostructure for Energy‐Efficient and High‐Accuracy Neuromorphic Computing,” Advanced Functional Materials 35, no. 48 (2025): 2509119, 10.1002/adfm.202509119.

[18] J. Yao , F. Lai , Y. Fan , et al., “Nonlocal Meta‐Lens With Huygens' Bound States in the Continuum,” Nature Communications 15, no. 1 (2024): 6543, 10.1038/s41467-024-50965-y. PMC11297327 39095407

[19] L. Zhang , Q. Wang , Z. Yu , et al., “Non‐Cascaded and Crosstalk‐Free Multi‐Image Encryption Based on Optical Scanning Holography Using 2D Orthogonal Compressive Sensing,” IEEE Transactions on Image Processing 33 (2024): 5688–5702, 10.1109/tip.2024.3468916. 39361468

[20] H. Liu , Y. He , J. Luo , et al., “Multimodal Actuation and Precise Control in Liquid Crystal Elastomer Optical Fiber Artificial Muscles,” Advanced Materials 37, no. 43 (2025): e05776, 10.1002/adma.202505776. 40801129

[21] W. Adi , S. Rosas , A. Beisenova , et al., “Trapping Light in Air With Membrane Metasurfaces for Vibrational Strong Coupling,” Nature Communications 15, no. 1 (2024): 10049, 10.1038/s41467-024-54284-0. PMC11579285 39567485

[22] W. Song , X. Liang , Q. Cheng , et al., “Full‐Color Waveguide‐Type Near‐Eye Displays With Huygens' Metasurfaces,” Advanced Optical Materials 13, no. 17 (2025): 2500096, 10.1002/adom.202500096.

[23] B. X. Wang , C. Xu , G. Duan , W. Xu , and F. Pi , “Review of Broadband Metamaterial Absorbers: From Principles, Design Strategies, and Tunable Properties to Functional Applications,” Advanced Functional Materials 33, no. 14 (2023): 2213818, 10.1002/adfm.202213818.

[24] H. Yu , Z. Liang , X. Shi , et al., “Long‐Infrared Broadband Polarization‐Sensitive Absorber With Metasurface Based on Ladder Network,” Advanced Optical Materials 12, no. 27 (2024): 2401104, 10.1002/adom.202401104.

[25] E. Hou , Z. Liang , X. Shi , et al., “Polarization‐Sensitive Metamaterial Absorber Based on the Composite Grating Structure in the Long‐Wave Infrared Band,” Optics and Laser Technology 175 (2024): 110676, 10.1016/j.optlastec.2024.110676.

[26] C. Li , G. Wang , M. Peng , et al., “Reconfigurable Origami/Kirigami Metamaterial Absorbers Developed by Fast Inverse Design and Low‐Concentration MXene Inks,” Acs Applied Materials & Interfaces 16, no. 32 (2024): 42448–42460, 10.1021/acsami.4c07084. 39078617

[27] Y. Yang , H. Kim , T. Badloe , and J. Rho , “Gap‐Plasmon‐Driven Spin Angular Momentum Selection of Chiral Metasurfaces for Intensity‐Tunable Metaholography Working at Visible Frequencies,” Nanophotonics 11, no. 17 (2022): 4123–4133, 10.1515/nanoph-2022-0075. PMC11501853 39635177

[28] Y. She , J. Ruan , D. Liu , et al., “Tunable High‐Performance Polarization Selectivity Empowered by Polarization‐Sensitive Fabry‐Perot Modes in One‐Dimensional Photonic Crystals Containing an Elliptical Metamaterial Defect,” Physica Scripta 100, no. 5 (2025): 055505, 10.1088/1402-4896/adc3d0.

[29] X. Tian , X. Qiu , H. Li , J. Lu , and C. Yang , “Dynamically Light‐Switched Polarization‐SENSITIVE Absorber Based on Semiconductor‐Incorporated Metamaterial Structure,” Optical Materials 149 (2024): 115139, 10.1016/j.optmat.2024.115139.

[30] X. Wang , C. Zhang , S. Rui , C. Wu , and F. Ma , “Multi‐Scale Material/Structure Integrated Elastic Metamaterial for Broadband Vibration Absorbing,” Materials & Design 238 (2024): 112705, 10.1016/j.matdes.2024.112705.

[31] J. Xue , C. Chen , S. Tian , et al., “Control and Optimization of Absorbing Behavior in Graphene‐Based Multiple Narrowband Metamaterial Absorber by Machine Learning,” Optics Communications 587 (2025): 131958, 10.1016/j.optcom.2025.131958.

[32] W. Zhou , Y. Zhang , X. Qin , et al., “A Dynamically Tunable Four‐Narrowband Terahertz Absorber Based on Dirac Semimetal for Perfect Absorption and High Sensitivity,” Optics Communications 584 (2025): 131845, 10.1016/j.optcom.2025.131845.

[33] T. Sang , H. Qi , X. Wang , et al., “Ultrabroadband Absorption Enhancement via Hybridization of Localized and Propagating Surface Plasmons,” Nanomaterials 10, no. 9 (2020): 1625, 10.3390/nano10091625. PMC7558930 32825058

[34] C. Tuo , J. Zhu , Z. Yi , B. Li , and S. Cheng , “Mid‐Infrared Broadband Triple‐Resonance Metamaterial Absorber Based on Triple‐Layer Silicon Nitride Structure,” Optics Communications 599 (2026): 132650, 10.1016/j.optcom.2025.132650.

[35] W. L. Barnes , A. Dereux , and T. W. Ebbesen , “Surface Plasmon Subwavelength Optics,” Nature 424, no. 6950 (2003): 824–830, 10.1038/nature01937. 12917696

[36] Y. Q. Ye , Y. Jin , and S. He , “Omnidirectional, Polarization‐Insensitive and Broadband Thin Absorber in the Terahertz Regime,” Journal of the Optical Society of America B‐Optical Physics 27, no. 3 (2010): 498, 10.1364/josab.27.000498.

[37] A. Tittl , M. G. Harats , R. Walter , et al., “Quantitative Angle‐Resolved Small‐Spot Reflectance Measurements on Plasmonic Perfect Absorbers: Impedance Matching and Disorder Effects,” ACS Nano 8, no. 10 (2014): 10885–10892, 10.1021/nn504708t. 25251075

[38] X. Sun , X. He , Z. Cai , and X. Hao , “Single‐Layer Chiral Metasurface for Circularly Polarized Light Detection,” Frontiers of Information Technology & Electronic Engineering (2025): 1454.

[39] D. Nguyen , D. Lee , and J. Rho , “Control of Light Absorbance Using Plasmonic Grating Based Perfect Absorber at Visible and Near‐Infrared Wavelengths,” Scientific Reports 7, no. 1 (2017): 2611, 10.1038/s41598-017-02847-1. PMC5454054 28572672

[40] X. Shi , Z. Qin , Z. Liang , et al., “Polarization‐Selective Absorptive and Transmissive Metamaterials,” Optics Express 30, no. 12 (2022): 20532, 10.1364/oe.456849. 36224795

[41] A. D. Rakic , A. B. Djurisic , J. M. Elazar , and M. L. Majewski , “Optical Properties of Metallic Films for Vertical‐Cavity Optoelectronic Devices,” Applied Optics 37, no. 22 (1998): 5271, 10.1364/ao.37.005271. 18286006

[42] E. Shkondin , O. Takayama , M. E. A. Panah , et al., “Large‐Scale High Aspect Ratio Al‐Doped ZnO Nanopillars Arrays as Anisotropic Metamaterials,” Optical Materials Express 7, no. 5 (2017): 1606, 10.1364/ome.7.001606.

[43] M. A. Ordal , R. J. Bell , R. W. Alexander , L. L. Long , and M. R. Querry , “Optical‐Properties of 14 Metals in the Infrared and Far Infrared—Al, Co, Cu, Au, Fe, Pb, Mo, Ni, Pd, Pt, Ag, Ti, V, and W,” Applied Optics 24, no. 24 (1985): 4493, 10.1364/ao.24.004493. 18224235

